# Electrodeless Heart and Respiratory Rate Estimation during Sleep Using a Single Fabric Band and Event-Based Edge Processing

**DOI:** 10.3390/s22176689

**Published:** 2022-09-04

**Authors:** Titus Jayarathna, Gaetano D. Gargiulo, Gough Y. Lui, Paul P. Breen

**Affiliations:** 1 The MARCS Institute, Western Sydney University, Westmead, NSW 2145, Australia; 2 School of Engineering, Design and Built Environment, Western Sydney University, Penrith, NSW 2750, Australia; 3 Ingham Institute of Applied Medical Research, Liverpool, NSW 2052, Australia; 4 Translational Health Research Institute, Westmead, NSW 2145, Australia

**Keywords:** edge computing, textile sensors, wearable sensors, wireless sensors

## Abstract

Heart rate (HR) and respiratory rate (RR) are two vital parameters of the body medically used for diagnosing short/long-term illness. Out-of-the-body, non-skin-contact HR/RR measurement remains a challenge due to imprecise readings. “Invisible” wearables integrated into day-to-day garments have the potential to produce precise readings with a comfortable user experience. Sleep studies and patient monitoring benefit from “Invisibles” due to longer wearability without significant discomfort. This paper suggests a novel method to reduce the footprint of sleep monitoring devices. We use a single silver-coated nylon fabric band integrated into a substrate of a standard cotton/nylon garment as a resistive elastomer sensor to measure air and blood volume change across the chest. We introduce a novel event-based architecture to process data at the edge device and describe two algorithms to calculate real-time HR/RR on ARM Cortex-M3 and Cortex-M4F microcontrollers. RR estimations show a sensitivity of 99.03% and a precision of 99.03% for identifying individual respiratory peaks. The two algorithms used for HR calculation show a mean absolute error of 0.81 ± 0.97 and 0.86±0.61 beats/min compared with a gold standard ECG-based HR. The event-based algorithm converts the respiratory/pulse waveform into instantaneous events, therefore reducing the data size by 40–140 times and requiring 33% less power to process and transfer data. Furthermore, we show that events hold enough information to reconstruct the original waveform, retaining pulse and respiratory activity. We suggest fabric sensors and event-based algorithms would drastically reduce the device footprint and increase the performance for HR/RR estimations during sleep studies, providing a better user experience.

## 1. Introduction

Continuous heart rate (HR) and respiratory rate (RR) monitoring is an essential part of sleep monitoring applications. HR and RR variation analysis during sleep could reveal the quality of sleep [1], the recovery efficiency of professional athletes [1] or the general wellbeing of a person, which may directly affect quality of life [2,3]. Generally, sleep occurs in a controlled and predictable environment where the subject is relatively still compared with during the daytime. However, the wearability of HR and RR monitoring devices requires optimization for comfort and long-duration overnight use.

Conventional methods of monitoring HR and RR during sleep are well-established. Polysomnography (PSG), the gold standard of sleep monitoring, uses either electrocardiogram (ECG) or photoplethysmography (PPG) for HR capture [4]. RR is conventionally captured with respiratory inductive plethysmography (RIP) belts [5,6], nasal pressure transducers [7], thermal sensing devices or acoustic methods [8]. Advancements in material science and consumer/researcher interest in wearable technologies have led to the production of a variety of wearable devices enabling heart rate and respiratory rate with smaller, more comfortable power-efficient devices. Some of these implementations are summarized in Table 1, with a focus on the capability to monitor HR and RR, the technology used, and how the sensor is applied in the wearable setting.

An unobtrusive, noncontact option of HR/RR measurement has its advantages, disadvantages, and limitations. Ideally, these sensors are placed away from the user. The distance from the user to the sensor location should be more than arm’s length, or hidden behind (such as a mattress, pillow or bed sheet) to avoid being a hindrance to the user, otherwise, it could degrade the original purpose and advantage. The simplest and most intuitive option is to use audio/visual sensors (i.e., microphones or cameras). Audio/visual methods are studied in tightly controlled environments where the authors reported accurate breath detection results [9]. The reliability of audio/visual methods drops significantly when the subject is standing [9] or in dark or noisy environments. In a dark environment, infrared or thermal cameras could be used as an alternative, however, these sensors are extremely expensive and only suitable in highly constrained scenarios where the sensor cost is justified. One experiment conducted with preterm neonates [10] to monitor respiration reported a root mean square error (RMSE) of 4.15±1.44 breaths/min and better RMSE with healthy adults 0.31±0.09 breaths/min. Another experiment conducted in an infrared illuminated room with an infrared camera reported a 3.4% mean percentage error for RR and 5% mean percentage error for HR calculation using independent component analysis [11]. These visual methods could be unfavorable due to privacy reasons as well.

Respiratory measurements from depth/time-of-flight sensors can be recorded when the sensor is mounted a few meters away from the user. One experiment conducted with Microsoft Kinect V2 with automated region-of-interest selection reported RR calculation within an error of 1±3.7 breaths/min [12]. Another experiment that uses both Microsoft Kinect and thermal cameras shows a good agreement between the calculated RR between two sensors [13].

Radar-based HR/RR measurement has become popular during the past few years due to declining sensor cost and the high utilization of radars in the automotive industry resulting in wider availability of sensors and algorithms. A study with 12 subjects conducted with a Doppler radar sensor to capture respiration and pulse shows good results in the supine position, while seated subjects showed degraded agreement with ground-truth [14]. In another experiment, Wavelet Independent Component Analysis was used to retrieve HR and RR [15], and the authors reported HR detection with an RMSE of 1.36 beats/min across 12 subjects. Once the sensor waves are focused, radar sensors could produce a fairly accurate estimation of HR/RR with proper processing algorithms. Radar sensors are not useful when the subject is moving across the field of view, if the subject is close to another person, or if the sensor requires battery-powered portable operation in long-term monitoring applications. Force- or pressure-based sensors mounted under a sleeping mattress/bed is another interesting approach tested in [16,17,18,19]. These solutions provide accurate readings for a single subject but are not suitable when used in a shared bed.

Fibre Bragg Gating (FBG)-based strain sensors have also been used in research to detect RR/HR [20], however, this method demands optical interrogator devices which are unsuitable in portable, low-cost solutions. FBGs response shifts with temperature, and the authors in [21] attempted to utilize this property to build a low-cost solution using a photo-detector without an optical interrogator. The authors report the estimated cost of the system is around USD 5000. However, FBGs are very useful in harsh environments with high electromagnetic interference where there are minimum alternatives [22,23].

Flexible electroactive materials demonstrate excellent potential to provide comfortable physiological monitoring at scale. However, current approaches share some common weaknesses in how they are applied, especially for sleep monitoring. Devices that require direct skin contact have the disadvantage of requiring thorough testing for long-term skin compatibility for humans, with the possibility that a group of people will react to the sensor material. Furthermore, adhesive-type skin contact sensors are uncomfortable to wear and remove, need frequent changing in long-term applications, and possibly cause skin irritation or skin damage [24,25]. For long-term monitoring, non-skin-contact sensors would be preferable. Fabric-based sensors, such as conductive fabric or capacitively coupled fabric electrodes, could be of further appeal to users if integrated into regular clothing as “invisible” sensors. The lack of rigid and firm body contact (e.g., electrode adhesive) makes them more likely to be impacted by body movement artifacts. Furthermore, capacitively coupled electrodes require more complex instrumentation such as filters, impedance matching circuits, and instrumentation amplifiers [26,27], which results in comparatively power hungry or larger devices. By comparison, electro-resistive fabric requires straightforward instrumentation to measure resistive changes using a single current source and an ADC. Vendors such as Texas instruments offer 24-bit high-precision ADCs with integrated, programmable constant current sources in a 3.5 mm × 3.5 mm package [28], making the full instrumentation circuit achievable in a single 12.25 mm ${}^{2}$ integrated circuit.

**Figure 1:**
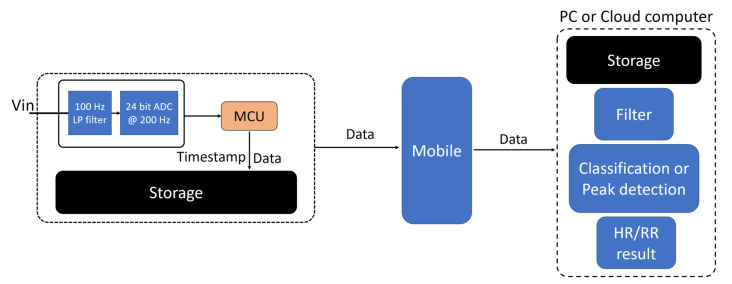
Typical architecture of an embedded data acquisition system showing a single channel input.

Another important factor often overlooked is that post-monitoring data analysis does not focus on the behavior of the raw data when the recording time is longer—instead, the analysis focuses on the change in derived parameters. For example, a full night sleep test analysis does not focus on the shape of the waveform of every respiratory or heart rate measurement. Instead, the analysis would be based on how the derived respiratory rate and heart rate vary with time. Due to this phenomenon, the ratio of useful information to available data is meager. This ratio is often between hundred-to-one to thousand-to-one depending on the target parameter and the sample rate. A typical data acquisition approach for a connected embedded system is shown in Figure 1 and exemplifies this issue. An internal microcontroller unit (MCU) captures and stores raw data to local memory with a timestamp. When the data acquisition period ends, data are transferred to an end device through either wired or wireless communication. Some devices will store data to ensure data integrity while transferring data to a wirelessly connected device in real time. Typically, a mobile device is used to relay raw data to an end computer unit for processing. While this method is relatively simple to implement, when the sampling rate is high, and higher precision is required, direct data acquisition generates a large amount of data.

For example, if a 200 Hz sampling rate is used, a single channel of 24-bit precision data with 32-bit timestamps would result in 38.45 MB of data for an 8-hour recording. The data size increases proportionally with the number of channels and sample rate. Storing and transferring a large amount of data wirelessly to a mobile phone either requires a longer time using low-power wireless methods (e.g., Bluetooth low energy (BLE) or near field communication (NFC)) or requires higher power for high-throughput protocols (e.g., WiFi). While data storage components have become cheaper, the power required to store/transfer data has not decreased significantly. Higher storage/bandwidth requirements pose a significant challenge to optimizing the size, cost, complexity, and power budget in small vital monitoring systems.

This work aims to solve two challenges: (1) provide a comfortable vital monitoring experience and (2) reduce the amount of raw data handling, hence reducing the storage and power requirements of the overall system. We hypothesized that DC-polarized, electrically conductive fabric-based morphic sensors could acquire both respiration and heart rate information from a single unit in a low-activity environment. Using a single sensor/channel for two vital parameters benefits the user from a comfort perspective but also significantly reduces data storage/transfer requirements. We further postulated that by using a novel event-based processing algorithm, we could discard most of the redundant data and accurately capture heart rate (HR) and respiratory rate (RR) at the micro-controller level. We hypothesized that this event-based approach would allow raw data to be processed into useful events that estimate beat-to-beat heart rate and breath-to-breath respiratory rate in real time.

Furthermore, we speculated that with the event-based approach, we could process and transmit data using substantially less power than transmitting/storing the raw data. Since the power requirement of the device directly affects device size and recharge frequency, such an event-based algorithm would be of substantial utility in wearable physiological monitors and edge computing more broadly. The novel event-based data pipeline we propose aims to process long-term recordings more efficiently in edge devices. Instead of storing and streaming raw samples from the morphic sensor, we generate events at the MCU where only the critical information is retained. The events capture only significant data oscillations. Often, these significant and periodic wave oscillations correlate with the peaks of respiration and pulse information. This paper explains the design and instrumentation of the sensor and the implementation and evaluation of event-based RR and HR estimation algorithms in edge devices.

## 2. Materials and Methods

### 2.1. Sensor Design

**Figure 2:**
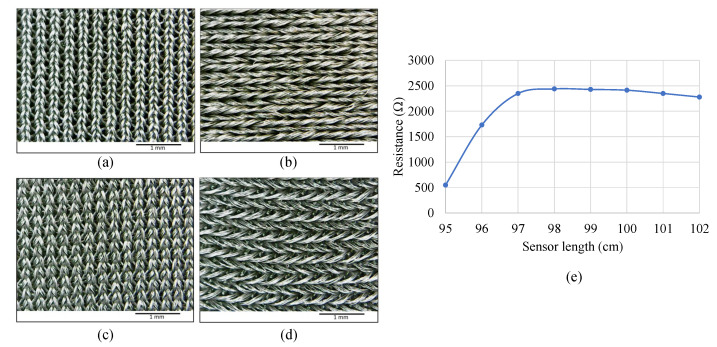
Sensor fabric and strain–resistance curve. (**a**) Unstretched front view, (**b**) unstretched back view, (**c**) stretched front view, (**d**) stretched back view, and (**e**) sensor stretch vs resistance measurement showing a nonlinear relationship.

The chest-worn sensor is based on a highly ionic, silver-plated nylon elastic fabric manufactured by Holland Shielding (Dordrecht, Netherlands) [38]. The double-direction knit pattern, as shown in Figure 2a,b, allows the fabric to stretch in both horizontal and vertical directions. The fabric has a different weaving structure on the front and backside, so that during the horizontal stretch, the back threading becomes looser (Figure 2d) while the front threading becomes tighter (Figure 2c). The tightness of threads induces an increase in conductivity and the loosening of threads introduces increased resistivity. When the fabric is cut into thin strips, the fabric strips behave like stretch sensors by changing resistivity. However, the stretch length vs resistance is not linear, as shown in Figure 2e. The nonlinearity of the fabric sensor used in our work is studied in detail in [39,40], where the results indicate the coating choice could increase nonlinearity. Nonlinearity properties of knitted fabrics are well-observed and characterized for different usages. Similar properties are observed for Electrolycra (Mindsets Ltd., Essex, UK) fabric [41,42] and EeonTex (Eeonyx Corporation, Pinole, CA, USA) [43]. Nonlinearity could be mitigated by exploring different cut patterns [44], using different coating materials and multiple coating techniques [45,46,47], curve fitting, or simply limiting the stretch to the linear region.

Nonlinearity becomes an advantage in HR/RR estimation with a single sensor. Generally, cardiac events are very low-amplitude compared with respiration, requiring a high sensitivity response from the sensor. As we intend to capture both respiration and cardiac activity from the same sensor, and respiratory wave requires a high stretch, a linear sensor could pose instrumentation difficulties for such a high dynamic range. The inherently low responsiveness at higher stretches simplifies instrumentation by naturally focusing the dynamic range in the cardiac response region.

**Figure 3:**
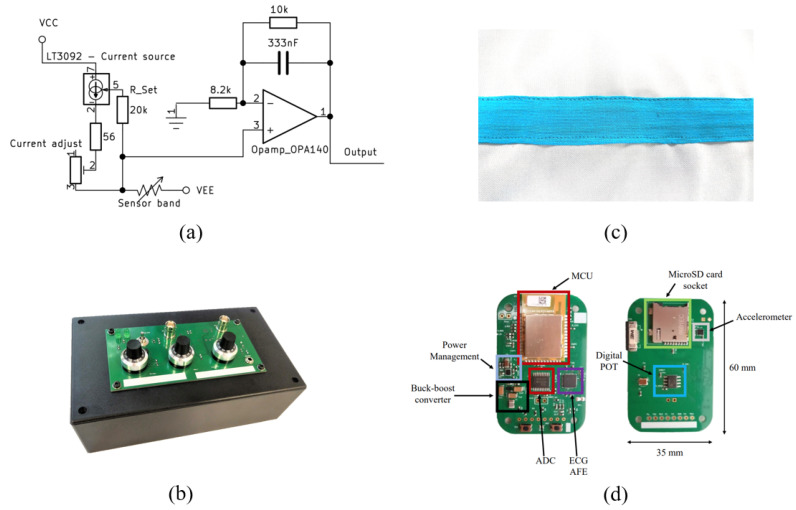
(**a**) Sensor instrumentation schematic view, (**b**) the developed PCB for bench tests, (**c**) the fabric sensor bands enclosed to kinesiology tape and sewn into the t-shirt fabric, and (**d**) proposed wearable data acquisition unit.

It should be noted that this linearity error would be a problem if the sensor were to be used in respiratory volume measurement; however, this is not relevant for our target HR/RR estimation. The sensor band is manufactured by cutting a 3 mm × 1000 mm fabric strip and concealing the fabric in U configuration using kinesiology tape (Figure 3c).

### 2.2. Sensor Instrumentation

A custom PCB was developed to acquire the signal from the fabric sensor. Since the fabric acts as a variable resistor under stretch, when polarized under constant current, the sensor produces a voltage output that could be either amplified or directly measured. An LT3092 programmable current source is used to generate the constant current, and the resulting voltage is fed through a noninverting filter amplifier circuit. The LT3092 current source can be controlled using a variable resistor to adjust the current. Figure 3a shows the schematic overview of the circuit, Figure 3b shows the final product used for testing the band, and Figure 3d shows the proposed wearable solution developed with additional ECG, accelerometer sensors for data verification purposes. Since the sensor evaluation is conducted in a bench setup, the Figure 3b PCB is produced with manual variable resistors to adjust the settings easily.

### 2.3. Experiment Setup

The experiments focus on evaluating the sensor in subjects staying in a stationary position. An example scenario would be sleep monitoring, aged/movement-restricted patient monitoring or infant monitoring. The sensor bands are worn in a supine position across the chest just over the heart such that the bands could capture the volume change in the chest due to respiratory movement and cardiac activity. The change in volume due to cardiac activity is much lower amplitude compared with respiratory volume; therefore, the pulse will always be superimposed onto the respiratory waveform. We recorded a 12 min recording from a healthy male (28 years) participant and used these data to develop the event-based HR/RR algorithm. We subsequently evaluated the performance of the algorithm in all three event-based architectures proposed. Next, we collected another data set from the same participant on a different occasion and used these data as the verification dataset.

### 2.4. Event-Based Processing Architecture

**Figure 4:**
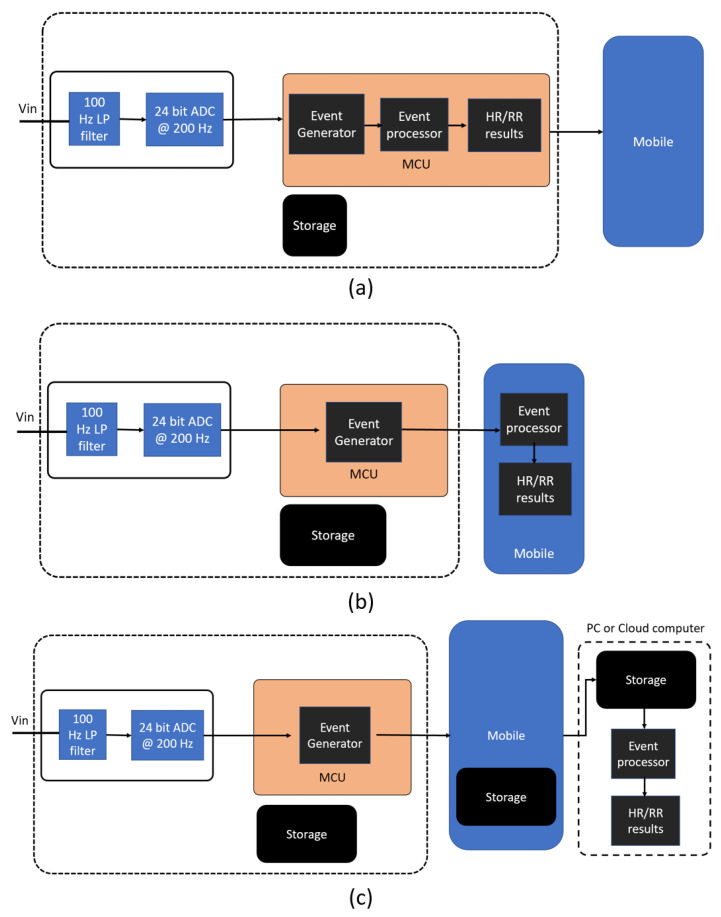
The abstract view of the event-based data processing flows in (**a**) event processing at MCU, (**b**) event processing at a mobile device, and (**c**) event processing at a PC or a cloud computer.

The event-based data processing follows a different architecture to a conventional setup, as shown in Figure 1. The idea is to reduce the computational complexity by converting raw data to useful data and push the processing toward edge devices. The event-based processing is demonstrated in three situations, but where the MCU is the event generator in all cases. The events generated at MCU could be either processed on (1) MCU itself to produce HR and RR estimations, (2) transferred to a mobile phone via a wireless method to process events at the mobile device or (3) relayed via a mobile platform to a PC or cloud computer for post-processing. We hypothesize that method (1) and (2) show advantages in power efficiency and real-time processing; however, they requires low-level custom algorithms. Method (3) is generally not suitable for real-time data analysis; however, it benefits from a great variety of available tools for post-processing data. Figure 4 shows the data flow in these three situations.

A Biopac AMI100D amplifier input module was used to capture data from the instrumentation circuit into a PC with a 10 kHz sample rate. The data are imported into MATLAB for analysis and filtered using a second-order, anti-aliasing Butterworth filter at 100 Hz cut-off frequency. The data were re-sampled to 200 Hz after filtering. As the ground-truth measure for the pulse recording, a single-channel ECG waveform is acquired at 10 kHz simultaneously. During the analysis, the ECG data were also re-sampled to 200 Hz. A Biopac ECG100C amplifier was used to capture the ECG waveform simultaneously.

## 3. Event-Based Algorithm Design

### 3.1. Raw Data Waveform Observation and Event Generation

**Figure 5:**
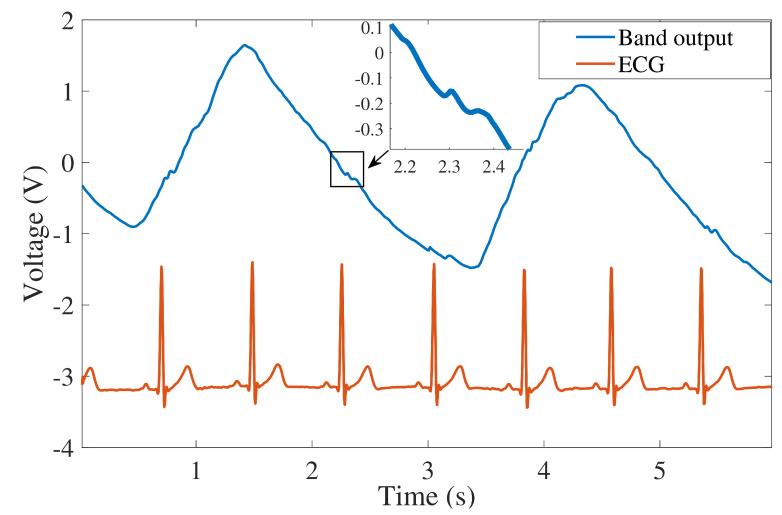
An example waveform from the sensor band showing the respiratory and pulse signal with corresponding ECG. Inset: zoomed-in view of the pulse.

An example waveform from the acquired signal is shown in Figure 5 and demonstrates a visible respiratory peak with smaller pulse peaks in synchrony with the ECG ground-truth. The inset shows a zoomed-in view of the pulse. Initial observations show the band captured the pulse pattern consistently; however, the signal height is small compared with the respiratory signal, as expected. For example, the peak-to-peak pulse amplitude shown in Figure 5 (inset) is 84 mV, while the respiratory wave shows 3120 mV peak-to-peak amplitude. The ratio between respiratory and pulse amplitude is 37.15, equivalent to a 31 dB difference.

An event generation algorithm based on delay–compare–integrate operations was explored to extract information of interest from the raw signal. The data Y(n) were compared with Y(n+p), where *p* is the delay. A compromise delay of ten samples (50 ms) was used as initial experiments found shorter delays produced many false positive events due to high-frequency noise, while longer delays produced more false negatives by ignoring pulse information.

An event signal V(n) was generated as follows:

(1) $$V\left(n\right)=\left\{\begin{array}{cc} 1, & Y(n+p)<Y(n)\\ 0, & Y(n+p)\geq Y(n) \end{array}\right.$$

The cumulative sum C(n) of each event signal is summed while V(n) is positive. Each falling edge of V(n) resets the cumulative sum to zero

(2) $$C\left(n\right)=\left\{\begin{array}{cc} V(n)+V(n-1), & V(n)=1\\ 0, & V(n)=0 \end{array}\right.$$

**Figure 6:**
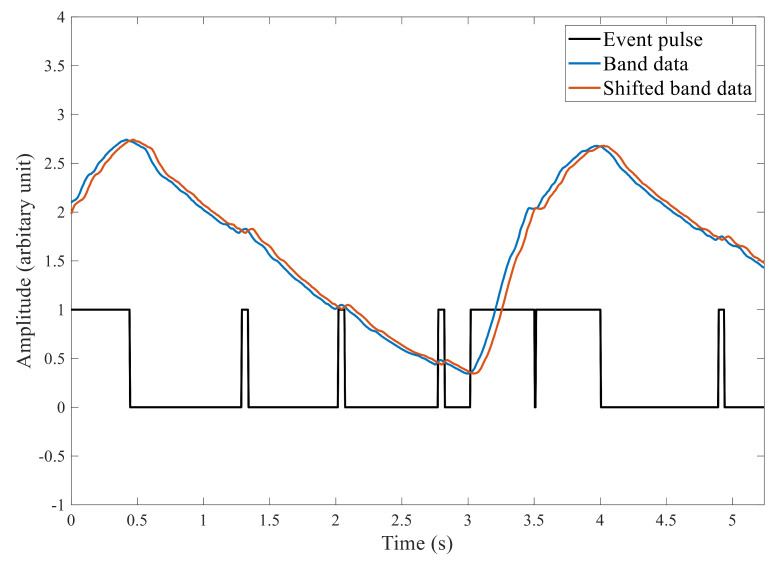
The output of the event generation step showing the original band signal (Y(n)), shifted band signal (Y(n+p)), and the event signal (V(n)) represented as binary values. Inverted event pulse during the inspiration (at 3.5 s mark).

**Figure 7:**
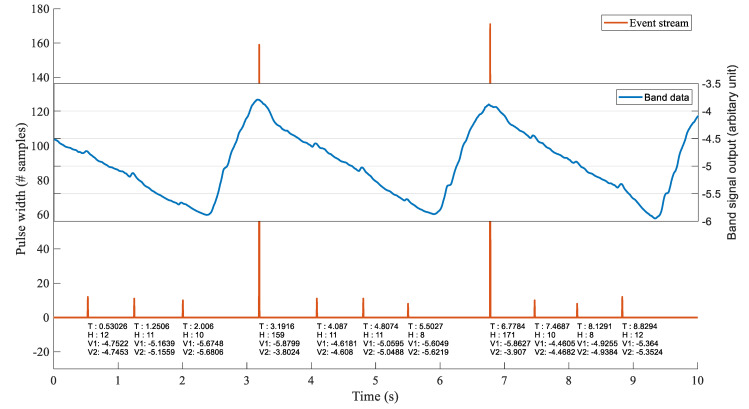
Events produced by the event-generating step of the algorithm. Each event has an associated timestamp (T), event period (H), band signal amplitude at the beginning (V1), and end of the event (V2).

As shown in Figure 6, the compare operation produces shorter high (V(n)=1) periods due to heartbeats and longer periods in response to respiratory peaks. The final cumulative sum C(n) prior to each reset (denoted *H*) is essentially the area of each event period. Ultimately, this method reduces all cardiac and respiration events to two numbers, the event period (*H*) and the timestamp at the end of the pulse. However, to reconstruct the entire signal of interest, the amplitude of the sensor signal at each positive and negative edge is included in the data structure. Thus, an event contains the following four fields, timestamp (*T*), event period (*H*), sensor amplitude at the positive edge (V1), and negative edge (V2). Each field is a 4-byte integer or floating-point number; therefore, each event produces 16 bytes of data. Figure 7 shows a visual representation of aligned events and raw data.

### 3.2. Algorithms for Event Processing

Ideally, the event processing algorithm would only require adaptive thresholding to separate cardiac and respiratory events and calculate HR and RR directly. However, real-world data produces an event stream from the delay–compare–integrate step with many false positives (FP) and false negatives (FN). Therefore, the event processing algorithm is responsible for:

- Separation of respiratory and cardiac events.
- Removal of events generated due to noise and artifact (false positive rejection).
- Compensation for missing events (false negative correction).
- Calculation of RR and HR.

#### 3.2.1. Separation of Respiratory and Cardiac Events

The event periods (*H*) related to respiratory peaks are significantly longer than those due to heartbeats. However, if a heartbeat occurs during inspiration, the respiratory event could be presented as two events, as shown in Figure 6 between the 3 and 4 s marks. Simple thresholding will present both events as respiratory events, resulting in a significant error in respiratory rate calculations. This behavior can be rectified before the separation of respiration and cardiac events.

An event stream of this kind shows two respiratory events separated by a very short time. Addressing these events requires combining two or more H>20 events. For each consequent pair of events (E1, E2), we check if fewer than 20 samples separate the events. If the sampling rate is *N*,

(3) $$\mathrm{Inter}\;\mathrm{Event}\;\mathrm{Period}\left(P\right)=\left(T_{2}-\frac{H_{2}}{N}\right)-T_{1}$$

If P×N<20, then we change the characteristics of the E1 and E2 events as shown in Table 2. After converting these occurrences into a cardiac and respiratory events, the thresholding function can separate the respiratory and cardiac events as outlined previously.

#### 3.2.2. Removal of Events Generated Due to Noise and Artifact (False Positive Rejection)

In this step, outliers are identified and removed based on timestamp data. The maximum respiratory rate is considered as 60 breaths per minute, while the maximum heart rate is considered 180 beats per minute (bpm). Within two events, the minimum allowable interval is defined using these two edge cases. When two subsequent events fall within this category, we reject one event only. The priority is given to the event with the larger *H* value.

#### 3.2.3. Compensation for Missing Events (False Negative Correction)

Due to the prominence of the respiratory signal, there is a very low chance of missing an actual respiratory peak. Therefore, false negative correction is applied solely for cardiac events where the likelihood of false negatives is much higher. Assuming the wearer is not missing heartbeats, we can actively estimate the number of missing beats in the data using local statistics. We utilize the fact that the number of heartbeats between any two other heartbeats is an integer value. For example, if three events show a calculated beat-to-beat rate of 70, 32, and 73 bpm, it is highly likely that a single beat was missed to produce the 32 bpm rate. We may confidently correct this to 64 bpm (closest integer multiplier to adjacent events), and the new instantaneous heart rate pattern would be 70, 64, 64, 73. The false negative correction part of the algorithm relies on actively injecting events to provide a reasonable estimate of the current heart rate using adjacent data instead of rejecting statistical outliers.

**Figure 8:**
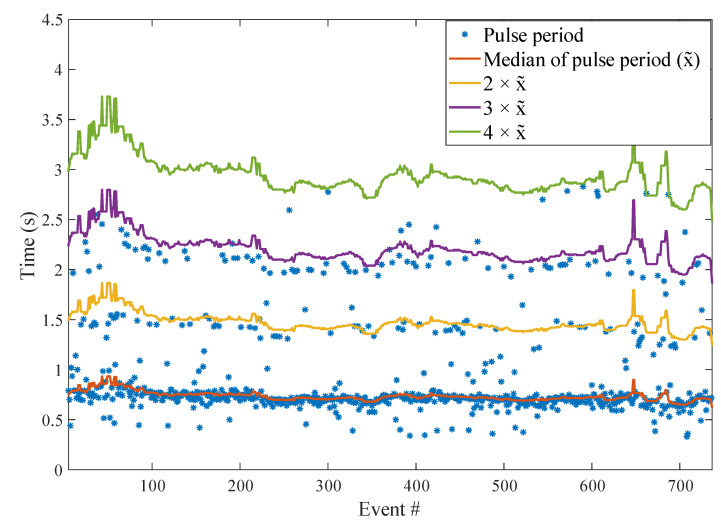
Cardiac event periods and 20-sample moving median value with its integer multiples.

Figure 8 presents a strong case for active compensation for missing beats. The graph shows the event periods for each subsequent cardiac event. We calculate the 20-sample moving median value ($\tilde{x}$) for each event and draw $2\times \tilde{x}$, $3\times \tilde{x}$, and $4\times \tilde{x}$ to visually represent the integer multiplication of $\tilde{x}$. The two, three, and four times median events occur when there are missing beats that result in a longer period. As shown in Figure 8, the missing events are clearly separable as groups and easily compensated for to produce an accurate HR estimation. Three alternative algorithms were explored to correct these false negatives. A conventional k-means algorithm tested the hypothesis that false negative correction would improve estimates of heart rate. A median-value-based algorithm and a bucketing algorithm were optimized and compared for minimal memory use and low processing power. The performance of the median value algorithm and bucketing algorithm were explored when implemented in the MCU, mobile device, and PC (Figure 4).

#### 3.2.4. Compensation for Missing Events Option 1—k-Means Clustering

k-means clustering in the MATLAB environment is used to cluster cardiac event periods into three groups. The initial seed values supplied to the algorithm were $\tilde{x}$, $2\times \tilde{x}$, and $3\times \tilde{x}$; 15 s, 30 s, and 60 s nonoverlapping windows were chosen. When the data are clustered, the group that has the most elements could be used to obtain the mean heart rate during the period by dropping other clusters; however, the aim is to compensate for missing beats. Instead of rejecting the remaining two clusters, each element is multiplied by an array of $\frac{1}{2}$, $\frac{1}{3}$, and $\frac{1}{4}$, and the dominant cluster mean is subtracted to calculate three error values. The absolute minimum error-producing multiplication is taken, and the event period is multiplied by the denominator of the lowest error-producing factor. The results are compared with the dominant cluster-based method to establish the benefit of nondominant clusters being used for false negative compensation.

### 3.3. Compensation for Missing Events Option 2—Median-Value-Based Algorithm

The goal of event-based architecture is to push data processing to the edge device without compromising battery life while simultaneously reducing memory requirements. The MATLAB solution that uses k-means clustering is not suitable for running on all three platforms (PC, Mobile, and MCU). Therefore, a lightweight algorithm based on median values was developed in the C programming language that requires less memory and computational power.

For each cardiac pulse event, the algorithm checks the median value $\tilde{x}$ for the past 30 cardiac events. Then, it generates the 1st, 2nd, 3rd, and 4th harmonics of $\tilde{x}$ by multiplying the median value by 2, 3, 4, and 5, respectively. The compensated period is obtained by dividing the event period by the median multiplication factor that produces the absolute minimum error. The median-value-based algorithm is simpler; however, it requires considerable floating-point operations to find a new median value for the past 30 events at each event it processes. Moreover, the algorithm cannot produce any HR estimation until the first 30 events have occurred.

### 3.4. Compensation for Missing Events Option 3—Bucketing Algorithm

The bucketing algorithm works similarly to a nonuniform histogram. An array of constant values is maintained, which keeps the bucket threshold values, and three dynamic arrays store event-related statistics.

- Bucket array = [ 0.4, 0.45, 0.5, 0.6, 0.7,0.8, 0.9, 1, 1.2, 1.6, 2, 2.4, 2.8, 3.2 ].
- Event array = [⋯] floating-point array of size 30 to store the last 30 cardiac pulse event periods.
- Mean array = [⋯] floating-point array the same size as the bucket array to maintain mean values for each bucket.
- Counter array = [⋯] integer array the same size as the bucket array to keep track of how many elements are in the corresponding bucket.

**Figure 9:**
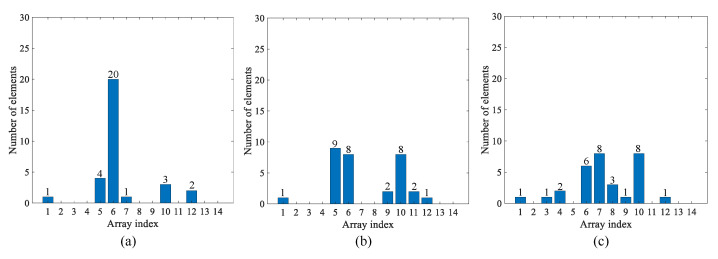
Example scenarios for counter array grouping strategy. (**a**) Single majority where one bucket has more than 20 elements. (**b**) Double majority where two adjacent buckets have the majority elements. (**c**) Triple majority where three adjacent buckets have the majority elements.

At each cardiac pulse event, the event period is calculated, and the bucket index is then found by comparing the event period to the values of the bucket array. For example, if the event period is 0.67 s, the bucket index is 5 (relates to 0.7 s). The arrays are updated by removing the first element from the event array and updating the corresponding mean and counter array values using the relevant index. The new element is appended to the event array, and the process repeats. By performing this removal and insertion operation, a record of events is maintained and grouped according to the reference bucket array. Since the counter array works as a histogram, we can easily differentiate the most frequent data (as cardiac pulse period) versus noise. Three separate majority measures are used (Figure 9) and prioritized in the following decreasing order to find the most frequent event period:

1. Single majority: If any element in the counter array is >20.
2. Dual combined majority: If the sum of two adjacent elements in the counter array is >15.
3. Triple combined majority: If the sum of three adjacent elements in the counter array is >15.

In case 1, the single majority value is returned. In cases 2 and 3, the mean value of adjacent elements is returned. If a majority cannot be found, the last valid HR value is returned.

After the algorithm finds a majority group with index *i*, it calculates the mean period using i−1, *i* and i+1 buckets. This allows the algorithm to capture oscillations of HR during the last 30 events which would be bucketed to adjacent indexes. Finally, the algorithm tries to compensate for possible missing beats. A clear example is shown in Figure 9, where a double majority case exists, and a group of possible missing beats are bucketed into indexes 9, 10, and 11.

After finding index *i* and the corresponding mean value, the algorithm scans the counter array forward to find any buckets that have more than three elements. If found, the algorithm checks the mean of the corresponding bucket and determines if the mean period of that particular bucket would fall into the 20 bpm range within already established HR (using majority mean) by dividing by 2 or 3. If it falls within the 20 bpm range, the corresponding elements are also included in the HR calculation. Similar to the median value algorithm, the bucketing algorithm has a pre-filling period where it cannot produce any HR output. The algorithm will wait until it has elements satisfying a single, double or triple majority, which could theoretically happen after 15 pulse events. However, in practice, a majority is typically found after 20–30 elements. The bucketing algorithm features a smaller number of floating-point arithmetic operations compared with the median-based algorithm.

### 3.5. Test Devices

A PC with Intel i7-6700K CPU running at 4.0 GHz with Ubuntu 14.04.5 LTS was used for benchmarking. The mobile device was a Samsung Galaxy S7 Edge (Seoul, South Korea) with Samsung Exynos Octa 8890, 2.6 GHz processor. The MCU tests were conducted using two platforms. A Texas Instruments (TI) CC2640 (ARM Cortex-M3, 48 MHz) was chosen as a test MCU without a floating-point arithmetic unit (FPU), and a TI CC2642 (ARM Cortex-M4, 48 MHz) was chosen as a test MCU with FPU support.

GCC 4.8.4 was used as the C compiler. An LLVM Clang cross-compiler provided by the Android NDK was used to compile the source code to the Android mobile platform. TI ARM compiler version 18 was used to compile the source code in both Cortex-M3 and Cortex-M4 platforms. Algorithm performance was measured in all three platforms using internal timers. In PC and mobile, the *gettimeofday()* function from the *sys /time.h* library was used, with a resolution up to 1 μs. In the CC2640 and CC2642 platforms, the internal timestamp service *Timestamp_get32()* was used, supporting up to 15 μs resolution. The power measurements are conducted with TI EnergyTrace technology [48] and presented as the average.

## 4. Results

First, the event detection performance of each algorithm step is presented incrementally based on the processing of offline data. Results from the k-means clustering approach are then presented followed by the results and benchmark testing of the two event-based algorithms (mean-value and bucketing) with comparisons when running on all three event-based processing architectures. Algorithms were compared considering processing time, algorithm performance, and power requirements.

### 4.1. Event Generation and the Effect of the False Positive (FP) Reduction Step

The shift and compare event generation method is compared with ground-truth ECG and respiratory wave data to assess the performance of the event generation procedure. The results are shown in Table 3 for each step and include event classification of HR and RR with and without false positive reduction. Respiration peak detection shows high sensitivity, precision and very low miss rate and false discovery rate. However, the pulse information shows a much higher miss rate (30%) but high precision, i.e., when a cardiac event is detected, there is a high chance that it is a real heartbeat. However, a high chance of missing events could negatively affect the overall performance of the algorithm. This result solidifies the requirement for a false negative compensation procedure which allows us to manage a high miss rate successfully.

### 4.2. HR and RR Estimation Using K-Means Clustering

**Figure 10:**
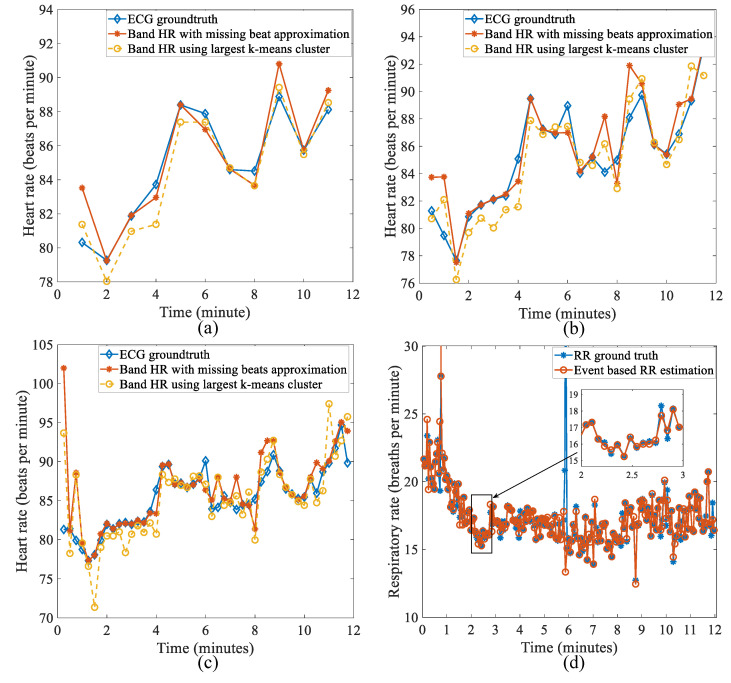
HR and RR estimation compared with ground-truth for the k-means algorithm. (**a**) HR estimation using a 1 min window. (**b**) HR estimation using a 30 s window. (**c**) HR estimation using a 15 s window. (**d**) Breath-to-breath RR estimation. The inset shows a closer view for one minute period.

The k-means algorithm is used for false negative correction, and the results are used as a baseline value to compare newly developed median value and bucketing algorithms. The k-means algorithm is applied to estimate HR after the FP reduction step. Breath-to-breath RR is directly calculated after FP reduction using timestamp information (no involvement of the k-means algorithm). The k-means algorithm uses 1 min, 30 s, and 15 s nonoverlapping windows to calculate HR. For each window, HR is estimated either by selecting only the dominant cluster or doing active missing beat compensation by approximation. Table 4 and Figure 10 shows the result compared with ground-truth calculated using ECG and respiratory wave peak detection (for RR). The results show that even a 15-s window could accurately estimate HR. Missing beats compensation reduces error and is more effective with shorter HR windows. However, the compensation technique increases the standard deviation of the error by a small margin. RR calculation does not use any false negative correction and compared well with ground-truth with an absolute mean error of only 0.18 breaths per minute.

### 4.3. HR and RR Estimation Using Median and Bucketing Method

Benchmark results of the two implementations of event processing algorithms running on a PC, a mobile phone, and MCUs (ARM Cortex-M3 and M4) are detailed. The benchmark results compare the computational requirements of the algorithms and are useful in determining the suitability of each algorithm implementation on an MCU/mobile in real time. The event generation part is only benchmarked on the MCU because there are no scenarios for the event-based architecture where the mobile device or PC act as the event generator (this would defeat the purpose of having the event-based architecture in the current context).

#### 4.3.1. Event Generation at MCU

The event generation step combines the delay, compare, and integrate steps to produce single events with a size of 16 bytes. A single event has four elements: Timestamp (*T*), event period (*H*), and Band signal amplitude at the beginning (V1) and end (V2) of the event. A total of 20,000 raw data points are loaded from a floating-point array and read sequentially. Reading data from an already constructed array isolates dependency on raw data read time, which can vary depending on the application (e.g., SPI bus speed and ADC acquisition time).

Table 5 shows the results from the two MCUs generating 146 events from the raw data samples. Processing time per sample is minuscule compared with the sample period (5000 μs). For comparison, if the MCU reads a 24-bit ADC via 6 MHz SPI bus, the data read time (4 μs) would still be longer than the event generation time of 2.7 μs.

#### 4.3.2. HR and RR Estimation Processing Time on PC, Mobile, and MCU for Median and Bucketing Algorithms

Event processing can occur on PC, Mobile or MCU. The event processing algorithm implemented in C is highly portable; we can run exactly the same source code with zero modifications on all three platforms. During the experiment, 12 min of data containing 1045 events are loaded from memory to calculate event processing time on each platform.

The median-value-based algorithm takes twice as much time on mobile devices compared with the PC implementation (Table 6); however, it is still extremely fast. The ARM Cortex-M3 MCU does not have a dedicated FPU and takes 1.37 ms on average to fully process a single event. The ARM Cortex-M4 MCU, which has an FPU, is four times faster and takes only 0.338 ms to process a single event. On the current dataset, the average event period (total time/number of events) is 688.995 ms, and the event processing takes a fraction of the event period to process data to comfortably achieve real-time performance.

The bucketing algorithm outperforms the median-value-based algorithm in terms of processing time (Table 6). It is 670% faster on PC, 590% faster on Mobile, 900% faster on the M3 MCU, and 680% faster on the M4 MCU at processing the same event data due to the reduced floating-point arithmetic requirements. When the event-based processing part of the algorithm runs on an MCU, it has to co-exist with the event generation process. The estimates in Table 7 show that both parts of the algorithm can run on the MCU with a comfortable margin to accommodate other background tasks. This example scenario includes the use of an external ADC to capture raw data with a 2 MHz SPI bus back to the MCU. We assume that the MCU is an ARM Cortex-M3 running the median-value-based algorithm to emulate the worst-case scenario.

### 4.4. HR and RR Estimation Results on PC, Mobile, and MCU for Median and Bucketing Algorithms

The two event-based algorithms, running on both PC mobile and MCU, were compared with ground-truth data. Since the same steps as the k-means algorithm are used (up to FP reduction) to calculate RR results in both implementations, there is no difference between RR results from the C implementation and results presented for offline MATLAB processing (Table 4). Moreover, since the same source code is operating on all platforms, there is no difference in the output for HR estimation between devices. The only difference is the HR estimation between the two algorithms. Instead of calculating the HR based on nonoverlapping windows, the HR is calculated at each HR event and estimated as the mean HR for the last 30 events. The output is smoothed by a six-sample averaging filter. The ground truth ECG HR is also produced by taking beat-to-beat HR and taking the mean for the past 30 beats.

**Figure 11:**
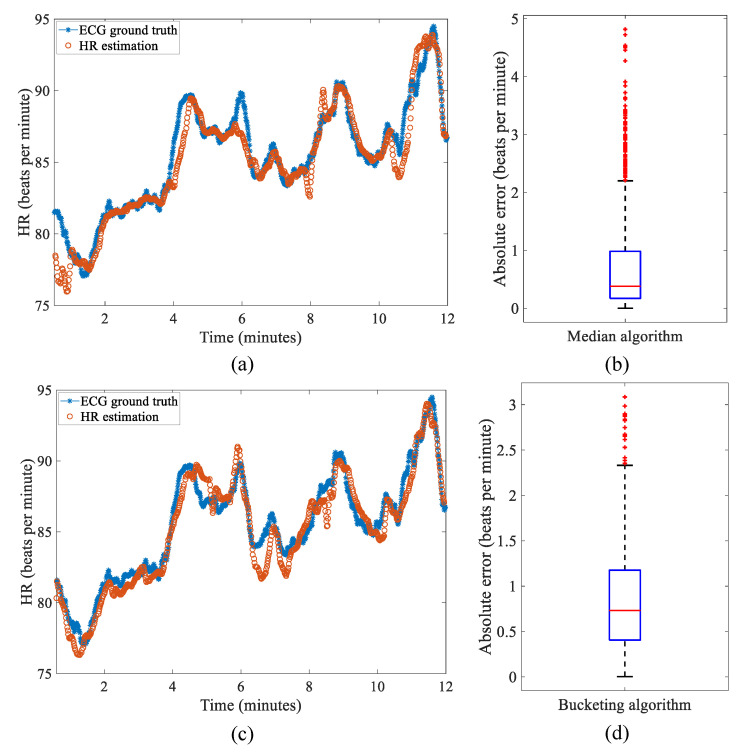
Results from the median-value-based algorithm and bucketing algorithm for HR estimation. (**a**) HR estimation compared with ECG ground-truth—median algorithm and (**b**) absolute error box plot. (**c**) HR estimation compared with ECG ground-truth—bucketing algorithm and (**d**) absolute error box plot.

Figure 11 shows the result from both the median-value-based algorithm and bucketing algorithm along with the corresponding absolute error shown as a box plot. The majority of error is less than one beat per minute, and the median-value-based algorithm is slightly more accurate than the bucketing algorithm as shown in Table 8.

### 4.5. Power Requirements during Continuous Operation of Event-based Algorithms

Regardless of the negligible processing time required, the event-based algorithm will add additional load to the MCU. Therefore, the power requirements of the MCU will increase slightly. However, the MCU is not only responsible for acquiring data. In a conventional system, the MCU needs additional subsystems to transmit and store the raw data. In this section, the power requirements to run the algorithm elements are computed. The power cost of running the event-based algorithm is compared with the power savings that may be realized by online processing and the inherent data size reduction that comes with that solution.

The data-handling pipeline may be defined in an additive fashion as follows:

- SYS0: Data acquisition at 200 Hz only.
- SYS0_G: Data acquisition at 200 Hz and generating events.
- SYS0_G_EVM: Data acquisition at 200 Hz, generating events and processing events using the median-value-based algorithm.
- SYS0_G_EVB: Data acquisition at 200 Hz, generating events and processing events using the bucketing algorithm.
- SYS0_W: Data acquisition at 200 Hz and transferring raw data wirelessly.
- SYS0_G_W: Running data acquisition at 200 Hz, generating events and transferring events wirelessly.
- SYS0_G_EVM_W: Running data acquisition at 200 Hz, generating events, process events using median-value-based algorithm and transfer results wirelessly.
- SYS0_G_EVB_W: Running data acquisition at 200 Hz, generating events, process events using the bucketing algorithm and transfer results wirelessly.

There is no scenario where SYS0 is useful; however, it was implemented to isolate the MCU power consumption of each element of the algorithm. As such, we can calculate the power requirement of the following elements:

- Power requirement of generating events
  POWG=SYS0_G−SYS0.
- Power requirement of median-value-based algorithm
  POWEVM=SYS0_G_EVM−SYS0.
- Power requirement of the bucketing algorithm
  POWEVB=SYS0_G_EVB−SYS0.

**Figure 12:**
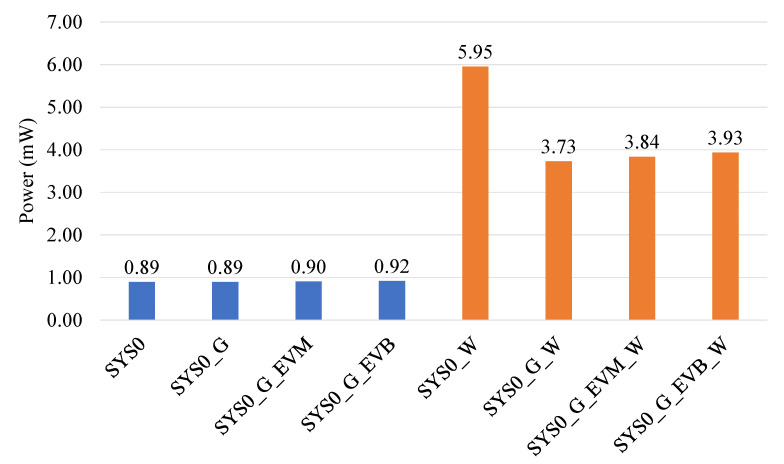
Power consumption for eight different data-handling pipelines running a combination of raw-data acquisition, event-based processing, and wireless data transfer on an ARM Cortex-M4 MCU.

Naturally, SYS0_G, SYS0_G_EVM, and SYS0_G_EVB all require more power than SYS0. However, a typical system would operate as SYS0_W. What is of interest from a practical perspective is to observe if the power requirements of SYS0_G_W, SYS0_G_EVM_W, and SYS0_G_EVB_W are less than SYS0_W. In other words, the question is to determine if it is more beneficial to (1) acquire and transfer data, (2) perform event generation and transfer events, or (3) run the full event-based algorithm and transfer results. Power consumption in all cases is shown in Figure 12 and Table 9.

Generating an event on the MCU costs 0.4 μW power, however, it gains a massive 2223.4 μW advantage overall due to the event-based architecture savings in data transmission volume. This is roughly 5500 times more efficient when considering algorithm optimization alone. Event generation and processing alone could save up to 33% power, even when running the full algorithm on the MCU and transmitting data to an external system. In this example, BLE v4.2 was used. As the sample period is 5 ms, and two samples can be embedded in a single BLE packet with the timestamp, a 12.5 ms connection interval was used.

### 4.6. Data Size Reduction with Event-Based Algorithms

The event-based algorithms were compared with the conventional approach on the basis of required data size (for transfer or storage depending on the application). The results for a 12 min dataset are summarized in Table 10. The original signal may be reconstructed if we choose to retain V1 and V2 values after processing the data.

Data size is reduced considerably in SYS0_G scenario, 1.45% of that required by SYS0. If we choose to compute the HR/RR on the MCU itself and discard the V1 and V2 values, we achieve a further data size reduction (0.73% of SYS0). Since the size of the data generated on the event-based algorithm depends on the HR and RR of the subject, the factor will vary depending on the subject. The results are presented for a subject with an average RR of 17.41 breaths/min and an average HR of 85.59 beats/min.

### 4.7. Band Signal Reconstruction Using Event Information

**Figure 13:**
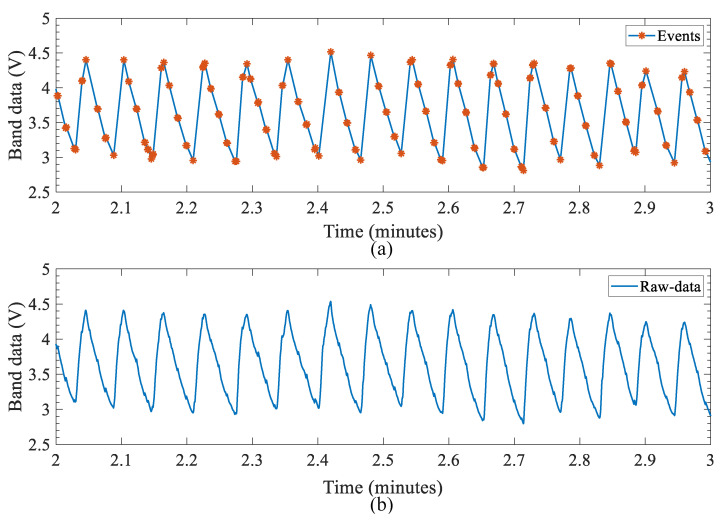
One-minute window of the data set showing the original waveform reconstructed using event-based data. (**a**) Waveform reconstructed using the event data. (**b**) Raw-data waveform.

Figure 13 shows a one-minute window from the dataset, where event-based data are used to reconstruct the original waveform. The events have retained all respiratory waveform information and retained some of the identified pulse information. By default, the event-based architecture retains the information to reconstruct and visualize the raw waveform for any verification purposes involved in the post-processing stage

## 5. Discussion

This work describes the use of a novel electrodeless and nonoptical flexible sensor that does not require direct skin contact to measure HR and RR. Furthermore, we present two event-based algorithms to extract respiratory and cardiac information in a manner suitable for edge IoT applications. The structure, methods, operation, and evaluation of these algorithms in terms of performance and power efficiency were compared with traditional methods and in multiple embodiments (MCU, mobile, and PC).

Results for RR calculation are accurate, with an absolute error of just 0.18±0.27 breaths/minute in breath-to-breath RR calculations. The sensor is sensitive to cardiac activity, and with the introduced delay–compare–integrate operation and the subsequent event generation, we observed high precision (95.15%), however, with a high event miss rate (30.82%). Three alternative methods to account for this miss-rate were implemented and tested. The k-means approach resulted in less than one beat-per-minute HR error with and without missing beat compensation (0.81±1 bpm vs. 0.83±0.61 bpm).

Event-based architectures were explored to determine if it is possible to push the computing task to the edge devices. Two event-processing algorithms were created, a median-value algorithm and bucketing algorithm, and evaluated on three proposed event-processing architectures (PC, Mobile, and MCU). Even the slowest processor among the three platforms tested (Cortex-M3) takes only 0.2% of the processing time available to compute the median-value-based algorithm and 0.02% of processing time available to compute the bucketing algorithm. The time taken to compute HR/RR on the MCU is insignificant compared with the other MCU operations involved in data acquisition. RR produced by these two algorithms is exactly the same as the previous approach, and therefore accurate up to 0.18±0.27 breaths per minute for the given dataset. The HR output is different from the k-means approach. The algorithms produce event-to-event HR and produce HR estimations with an absolute mean error of 0.81 bpm for the median-value-based algorithm and 0.86 bpm absolute error for the bucketing approach. HR estimations closely follow ground-truth data observed from simultaneous ECG recordings. Ultimately, the computation time and accuracy of these algorithms make them suitable for real-time processing on the MCU edge device.

The advantages of event-based data processing are evident when compared with a more conventional approach. Events are generated less frequently (about 130 times less) than raw data samples, requiring less frequent packet transfers and reducing the power budget required for wireless communication. The additional load on the MCU to generate and run the event-based algorithm is insignificant compared with the power efficiency achieved by the small bandwidth requirement of events or processed HR/RR values. If the MCU is generating events only, the additional power required to generate events is only 0.4 μW. However, if we add the wireless communication cost to transfer raw data, overall power consumption is reduced by 2223.4 μW. Even if the MCU is processing events, the power required is about 22.8 μW, however, a 2019.6 μW power reduction is achieved when wireless transmission costs are considered. A 70-to-140 times data size reduction is further achieved compared with raw-data size. We predict an even higher benefit when data storage power requirements are incorporated, a topic not explored in this paper. Ultimately, the reduced power and data requirements could lead to a smaller battery and smaller wearable solutions.

While there are some significant advantages of this type of sensor and event-based data processing approach, some weaknesses and limitations are of note. Since there is a high chance of missing a heartbeat when the band data is processed through the automated algorithm, our approach (flexible fabric bands on the chest) cannot be used to diagnose any conditions which may cause skipped beats or arrhythmia. Moreover, the sensor cannot be used to extract the cardiac pulse during significant movement. The body movement dramatically reduces the signal-to-noise ratio, and cardiac information is not easily distinguished from noise using this approach. Therefore, our sensor is only usable in low-activity monitoring scenarios (e.g., sleep and infants). Our dataset is used only as a proof-of-concept and as an example scenario to evaluate the performance of the algorithm and the sensor. Additional testing is required on different body sizes and ages to fully understand the usability of the sensor. However, the algorithms we proposed are general-purpose, and therefore could be used in any other sensor platform (ECG or PPG) with few modifications. We believe the event-based process architecture allows for greatly reduced data size and power consumption and enables the MCU to compute the required result at the edge.

## Figures and Tables

**Table 1:** Physiological monitoring wearables summarized based on measurement technology and sensor application method.

Study	Physiological Measurement		Measurement Technology	Application Method
	HR	RR		
Stephen P. Lee et al. [29] (2018)	**✓**	**✗**	Adhesive ECG electrodes	Attached to the skin
Insang You et al. [30] (2016)	**✓**	**✓**	Resistive elastomer	Attached to the skin
Roudjane et al. [31] (2018)	**✗**	**✓**	Wireless antenna attenuation	Sewn into the clothing
Presti et al. [32] (2019)	**✗**	**✓**	Flexible fiber Bragg grating	Sensor bands are worn over regular clothing
“Phyjama”, Kiaghadi et al. [33] (2019)	**✓**	**✓**	Resistive sensor fabric	Sensor patches are sewn to the underside of the clothing
Kim et al. [34] (2015)	**✓**	**✗**	Flexible RF resonator	The sensor is placed on the wrist close to the skin (no contact)
Rachim et al. [35] (2016)	**✓**	**✗**	Capacitively coupled fabric electrodes	Armband placed on top of regular clothing
Wu et al. [36] (2015)	**✓**	**✓**	Capacitively coupled fabric electrodes	Chest band placed on top of regular clothing
Ràfols-de-Urquía et al. [37] (2018)	**✓**	**✓**	Surface diaphragm electromyography	Electrodes directly attached to the skin

**Table 2:** Reassigning the event values for respiratory events when separated by a pulse event.

*E*1	*E*2
H=P×N	$H=H_{1}+H_{2}$
$T=\left(T_{2}-\frac{H_{2}}{N}\right)$	$T=T_{2}$
$V1=V1_{2}$	$V1=V1_{1}$
$V2=V2_{1}$	$V2=V2_{2}$

**Table 3:** Respiratory and cardiac event detection performance after event generation and false positive reduction.

	Algorithm Step	# of TP	# of FP	# of FN	Sensitivity (%)	Precision (%)	Miss-Rate (%)	False Discovery Rate (%)
**Respiration**	Event generation	206	3	2	99.03	98.56	0.96	1.43
**Respiration**	+FP reduction	206	2	2	99.03	99.03	0.96	0.96
**Pulse**	Event generation	721	102	301	70.54	87.60	29.45	12.39
**Pulse**	+FP reduction	707	36	315	69.17	95.15	30.82	4.84

**Table 4:** HR/RR calculation descriptive statistics (k-means algorithm for HR and simple thresholding for RR) showing mean absolute error and standard deviation (STD) of the absolute error.

	Window Size	Absolute Mean Error ± STD (Beats or Breaths per Minute)	
		No Compensation	With Compensation
	60 s	0.83±0.61	0.81±1.00
HR	30 s	1.37±0.85	1.05±1.43
	15 s	2.17±2.54	1.56±3.38
RR	NA (breath-to-breath)	0.18 ± 0.27	Not required

**Table 5:** Event generation time using ARM Cortex-M3 and Cortex-M4 MCUs processing 20,000 samples (100 s dataset). A total of 146 events generated.

Device	Total Time	Event Generation Time per Sample	Sample Period (at 200 Hz)	Processing Time per Sample Period
ARM Cortex-M3 (CC2640R2F)	53.8 ms	2.70 μs	5000 μs	0.054%
ARM Cortex-M4F (CC2642R1F)	40.5 ms	2.03 μs	5000 μs	0.041%

**Table 6:** Event processing time for PC, Mobile, and two ARM processors for 1045 events generated during a 12 min recording.

Device	Total Processing Time (ms)	Average Processing Time per Event (ms)	Processing Time per Average Event Period
Median Value-Based Algorithm			
PC	6.02	0.00576	0.000836%
Mobile	13.29	0.01272	0.001846%
ARM Cortex-M3	1428.4	1.36688	0.198387%
ARM Cortex-M4F	353.2	0.33799	0.049055%
Bucketing Algorithm			
PC	0.90	0.00086	0.000124%
Mobile	2.26	0.00216	0.000313%
ARM Cortex-M3	159.8	0.15291	0.022193%
ARM Cortex-M4F	52.8	0.05052	0.007332%

**Table 7:** Total estimated processing time to run the full algorithm in ARM Cortex-M3 MCU with an external ADC with 200 Hz sample rate and 2 MHz SPI clock.

Operation	Time	Comment
Raw data acquisition	12 μs	SPI runs at 2 MHz and external ADC samples parallel to the MCU process.
Event generation	2.7 μs	To process a single raw-data sample
Event processing	9.9 μs	Average process time divided across the number of samples.
Free time for context switching, interrupt handling, wireless communication, and other background tasks.	4975.4 μs	At 200 Hz, the time between samples is 5000 μs.

**Table 8:** Error statistics for hr estimation using median/bucketing algorithms.

Algorithm	Absolute Error: Mean (bpm)	Absolute Error: StDev (bpm)	Absolute Error: Median (bpm)
Median	0.81	0.97	0.38
Bucketing	0.86	0.61	0.73

**Table 9:** Additional power and current requirements of running the event-based algorithm on MCU.

	Event Algorithm	Power (μW)	Current (μA)
	SYS0_G	0.4	0.1
Compared with SYS0	SYS0_G_EVM	11.9	3.6
	SYS0_G_EVB	22.8	6.8
	SYS0_G_W	−2223.4	−673.8
Compared with SYS0_W	SYS0_G_EVM_W	−2116.0	−641.3
	SYS0_G_EVB_W	−2019.6	−612.1

**Table 10:** Data size comparison with and without running the event-based algorithm.

Use Case	Number of Elements	Data Size	Data Size with Raw Data Reconstruction
SYS0	143,832	1123.68 kB	N/A
SYS0_G	1045	16.33 kB	16.33 kB
SYS0_G_EVB & SYS0_G_EVM	1045	8.16 kB	16.33 kB

## Data Availability

Source code and sample data available on https://github.com/titusnkumara/eventbasedHRandRR (accessed on 8 August 2022).

## Abbreviations

The following abbreviations are used in this manuscript:

ADC	Analog-to-Digital Converter
BLE	Bluetooth Low-Energy
CPU	Central Processing Unit
DC	Direct Current
ECG	Electrocardiogram
FBG	Fibre Bragg Gating
FN	False Negatives
FP	False Positives
FPU	Floating Point Unit
GCC	GNU Compiler Collection
HR	Heart Rate
LTS	Long Term Support
MCU	Microcontroller Unit
NDK	Native Development Kit
NFC	Near Field Communication
PC	Personal Computer
PCB	Printed Circuit Board
PPG	Photoplethysmography
PSG	Polysomnography
RF	Radio Frequency
RIP	Respiratory Indictive Plethysmography
RMSE	Root Mean Square Error
RR	Respiration Rate
TI	Texas Instruments
TP	True Positive
Vcc	Voltage Common Collector
Vee	Voltage Common Emitter
